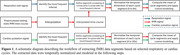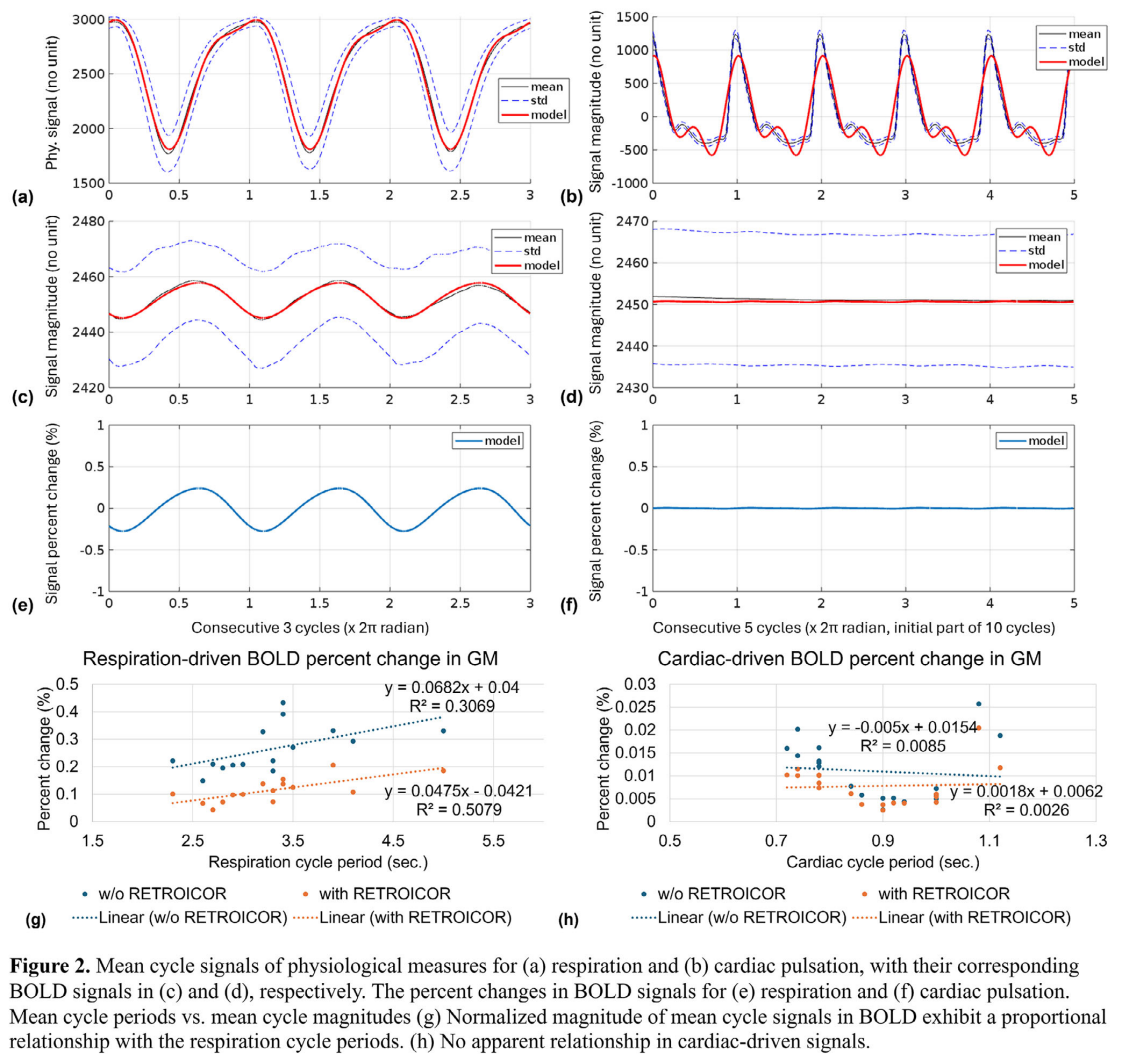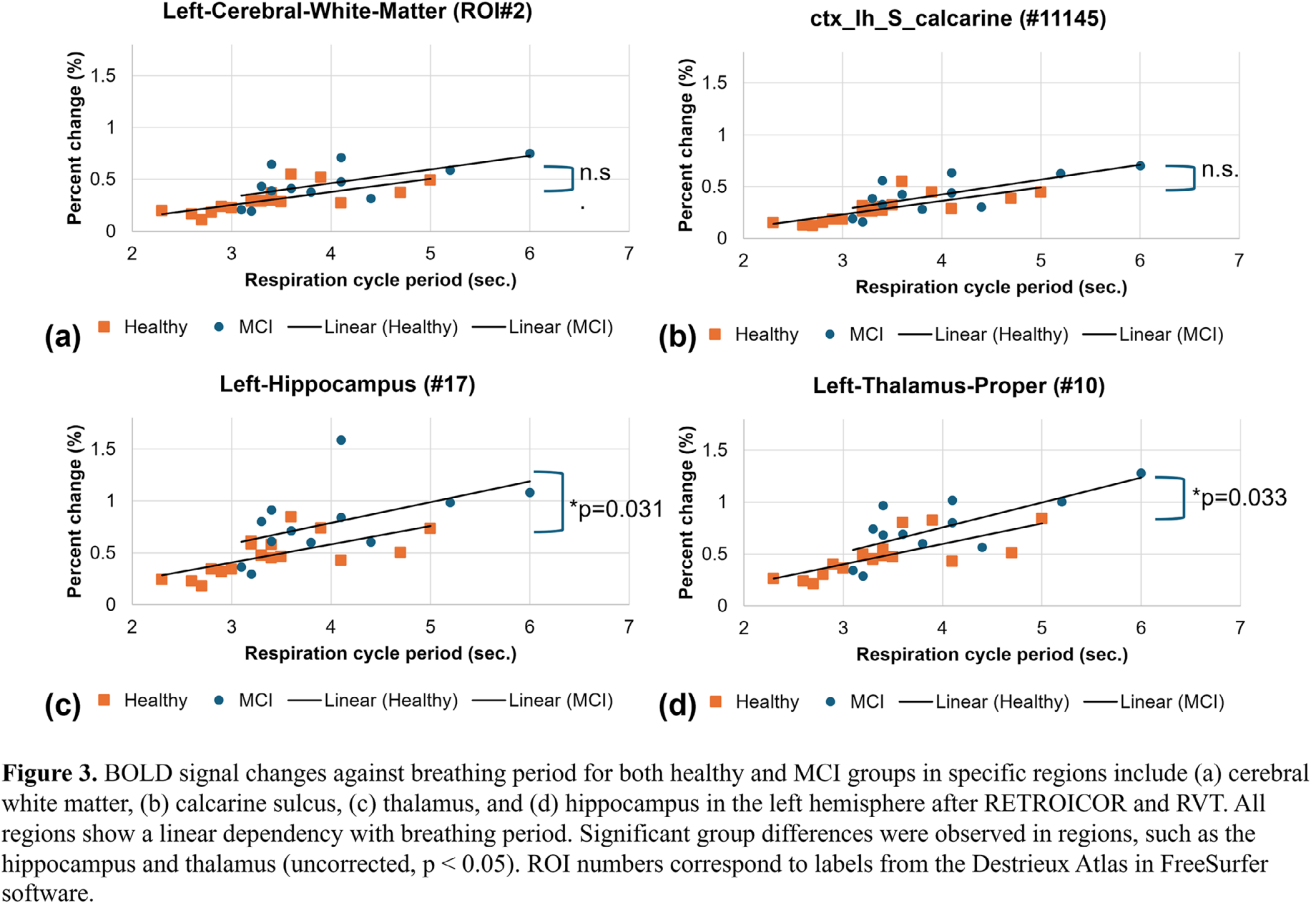# Breath‐Triggered Signals Observed in Resting‐State BOLD fMRI

**DOI:** 10.1002/alz70856_103789

**Published:** 2025-12-25

**Authors:** Daehun Kang, Paul H Min, Seokbeen Lim, Jeyeon Lee, Maria I. Lapid, Yunhong Shu

**Affiliations:** ^1^ Mayo Clinic, Rochester, MN, USA; ^2^ Hanyang University, Seoul, Korea, Republic of (South)

## Abstract

**Background:**

Respiration has traditionally been considered a source of artifact in functional MRI (fMRI) due to chest motion. However, the respiratory cycle also induces changes in local oxygen concentration, resulting in fluctuations in the fMRI signal. These breathing‐triggered signals (BTS) may reflect underlying oxygen metabolism. This preliminary study developed a method to extract BTS from resting‐state BOLD fMRI and examined differences between healthy and mild cognitive impairment (MCI) groups.

**Method:**

MRI scans were conducted under IRB approval on a compact 3T MRI system. Participants included two groups of 18 healthy volunteers (42.6±16.0 years) and 12 MCI patients (70.9±7.3 years). Each MRI scan included T_1_‐weighted anatomy imaging and multi‐echo resting‐state fMRI acquisitions. The preprocessing pipeline was implemented by AFNI and FreeSurfer. Figure 1 illustrates the workflow for extracting and averaging fMRI data based on respiratory or cardiac cycle windows. As depicted in Figure 2(c,d), the mean fMRI signals corresponding to the breathing or cardiac cycles were modeled using a low‐order Fourier series to assess their amplitudes. A linear mixed‐effects model was employed to examine the effects of group and breathing cycle period, using MATLAB's ‘fitlme’ function with the formula: BTS ∼ Period + Group.

**Result:**

As shown in Figure 2(e,f), breathing‐triggered BOLD signals were prominent, while cardiac‐driven BOLD signals were rarely observed. Figure 2(g) shows that global BTSs in the healthy group exhibited a linear relationship with the breathing cycle period, persisting even after artifact correction (RETROICOR for respiration and cardiac cycles and RVT). Figure 3 highlights significant regional differences between groups, particularly in the hippocampus and thalamus (*p* < 0.05, uncorrected), where BTS amplitudes were greater in the MCI group. Potentially, the detected BTS could provide some insights into the coupling between respiration and cerebral oxygen metabolism and potentially serve as an imaging biomarker to assess brain health. However, due to the limited number of samples, these findings should be interpreted with caution, and further analysis is needed.

**Conclusion:**

This preliminary study suggests that breathing‐triggered signals in resting‐state BOLD fMRI have the potential to serve as a noninvasive biomarker for early detection and monitoring of neurodegenerative conditions such as MCI.